# Protective operative techniques in radical hysterectomy in early cervical carcinoma and their influence on disease-free and overall survival: a systematic review and meta-analysis of risk groups

**DOI:** 10.1007/s00404-021-06082-y

**Published:** 2021-05-22

**Authors:** Johanna Kampers, E. Gerhardt, P. Sibbertsen, T. Flock, R. Klapdor, H. Hertel, M. Jentschke, P. Hillemanns

**Affiliations:** 1 Department of Gynecology and Obstetrics, Hannover Medical School, Karl-Neuberg-Str. 1, 30625 Hannover, Germany; 2 Faculty of Economics and Management, Leibniz University Hannover, Hannover, Germany

**Keywords:** Early cervical cancer, Hysterectomy, Minimally invasive, Operating techniques, Oncologic outcome, Uterine manipulator

## Abstract

**Purpose:**

Radical hysterectomy with pelvic lymphadenectomy presents the standard treatment for early cervical cancer. Recently, studies have shown a superior oncological outcome for open versus minimal invasive surgery, however, the reasons remain to be speculated. This meta-analysis evaluates the outcomes of robotic and laparoscopic hysterectomy compared to open hysterectomy. Risk groups including the use of uterine manipulators or colpotomy were created.

**Methods:**

Ovid-Medline and Embase databases were systematically searched in June 2020. No limitation in date of publication or country was made. Subgroup analyses were performed regarding the surgical approach and the endpoints OS and DFS.

**Results:**

30 studies fulfilled the inclusion criteria. Five prospective, randomized-control trials were included. Patients were analyzed concerning the surgical approach [open surgery (AH), laparoscopic surgery (LH), robotic surgery (RH)]. Additionally, three subgroups were created from the LH group: the LH high-risk group (manipulator), intermediate-risk group (no manipulator, intracorporal colpotomy) and LH low-risk group (no manipulator, vaginal colpotomy). Regarding OS, the meta-analysis showed inferiority of LH in total over AH (0.97 [0.96; 0.98]). The OS was significantly higher in LH low risk (0.96 [0.94; 0.98) compared to LH intermediate risk (0.93 [0.91; 0.94]). OS rates were comparable in AH and LH Low-risk group. DFS was higher in the AH group compared to the LH group in general (0.92 [95%-CI 0.88; 0.95] vs. 0.87 [0.82; 0.91]), whereas the application of protective measures (no uterine manipulator in combination with vaginal colpotomy) was associated with increased DFS in laparoscopy (0.91 [0.91; 0.95]).

**Conclusion:**

DFS and OS in laparoscopy appear to be depending on surgical technique. Protective operating techniques in laparoscopy result in improved minimal invasive survival.

**Supplementary Information:**

The online version contains supplementary material available at 10.1007/s00404-021-06082-y.

## Introduction

According to national and international guidelines, surgical therapy is recommended for early cervical cancer (FIGO Stadium ≤ IIA) [1]. Different surgical approaches have been established over the last century. However, randomized controlled studies evaluating the oncological outcome of the different approaches have been missing. Whereas the abdominal radical hysterectomy (AH) has been the method used for the longest period of time, according to reviews of mostly retrospective studies, it appears to be associated with a higher rate of morbidities, such as bladder dysfunctions, longer hospital stays or postoperative infections [2–4]. Also, systematic reviews have shown superiority of laparoscopic hysterectomy (LH) regarding inoperative blood loss, hospital stay and postoperative complications [5–7]. Additionally, these reviews reported similar oncological outcomes between LH and AH which led to the wide implementation of LH as a standard approach in early cervical cancer [5, 6]. In addition, meta-analyses comparing robotic minimally invasive (RH) to LH or AH, such as the ones by Park et al. [8], Zhou et al. [9] and Zhang et al. [10], could show a non-inferiority of robotic approaches regarding intra- and postoperative complications.

The publication of the LACC (Laparoscopic Approach to Cervical Cancer) trial in 2018, the first large multicenter randomized controlled trial comparing AH with LH, led to a drastic change of recommendations for surgical treatment [11]. The LACC study showed a significantly reduced overall- (OS) and disease-free survival (DFS) in the LH group (including robotic surgery) compared to the AH group. A reduction of perioperative complications in the LH group was not shown either.

The trial became controversially discussed due to its dramatic results concerning outcome of widely used laparoscopic techniques and contradicted the previous results of the metanalyses stated before. However, several retrospective studies published after the LACC trial showed significantly better outcomes for AH compared to LH and were in line with the LACC trial’s results. Furthermore, the recent meta-analysis of observational studies by Nitecki et al. found that minimally invasive radical hysterectomy was associated with an elevated risk of recurrence and death compared with open surgery [12].

A central point of discussion arose about the lack of standardization of the surgical procedure in this worldwide LACC trial [13]. Assumed risk factors for intraoperative tumor cell dissemination, such as the use of a uterine manipulator and the intraabdominal colpotomy, were postulated as not prerequisite for oncologic safe techniques in minimally invasive surgery.

Prior to the initiation of new prospective studies, a meta-analysis of previous studies should be conducted specifically looking on surgical techniques of laparoscopic radical hysterectomy to better understand factors that might influence oncologic and safety outcome.

## Materials and methods

The methods for this study were specified a priori based on the recommendations in the Preferred Reporting Items for Systematic Reviews and Meta-Analyses (PRISMA) statement [14].

### Search strategy

A systematic database research for studies comparing RH, LH and/or AH for the treatment of early cervical cancer via Ovid-Medline and EMBASE without restriction of the year of publication was performed. Search terms combined MESH-terms (uterine neoplasms) or Emtree headings and the related terms “cervical cancer”, “uterine cancer”, “cervical neoplasm”, as well as “laparoscopic surgery”, “hysterectomy”, “Wertheim operation”, “Robotics,” and “robotic-assisted surgery”.

### Study selection

**Fig. 1:**
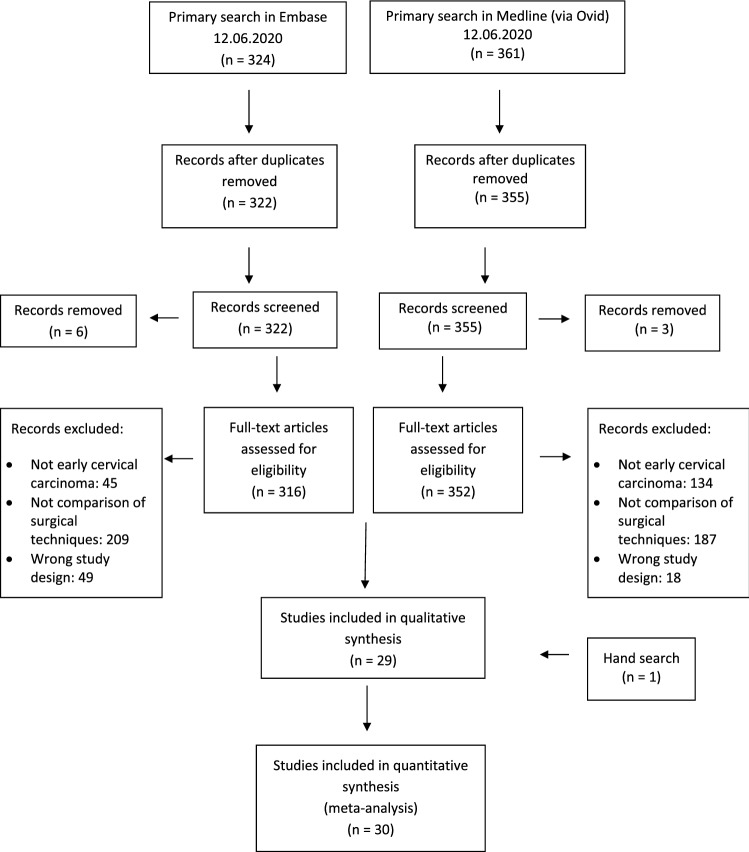
PRISMA flow chart

Study selection was done independently by JK and EG. In case of conflicting opinions, PH decided about inclusion or exclusion. The inclusion criteria were adapted to the inclusion criteria of the LACC trial [11] and specified in (1) studies that included patients with early cervical cancer FIGO IA1, IA2, IB1, IB2, IIA1, (2) comparative studies between RH or AH or LH, (3) studies that reported at least one outcome of interest, and (4) published original, peer-reviewed articles. Only studies with complete publication of all results were considered. Non-original studies, animal or preclinical trials, abstract-only publications, reports in a language other than English or German and duplicates were excluded. All reasons for exclusion are mentioned in the Preferred Reporting Items for Systematic Reviews and Meta‐Analyses flowchart (Fig. 1). One study already presented at ESGO congress prior to the systematic research was added by hand search upon publication. Three single-armed trials from the primary search were added to the final analysis.

The algorithms used for primary search as well as the full list of search results can be found in the supplementary items. If possible, the authors of studies that were only published as congress abstracts were tried to be contacted via email and asked to provide their data.

### Data extraction and quality assessment

**Fig. 2:**
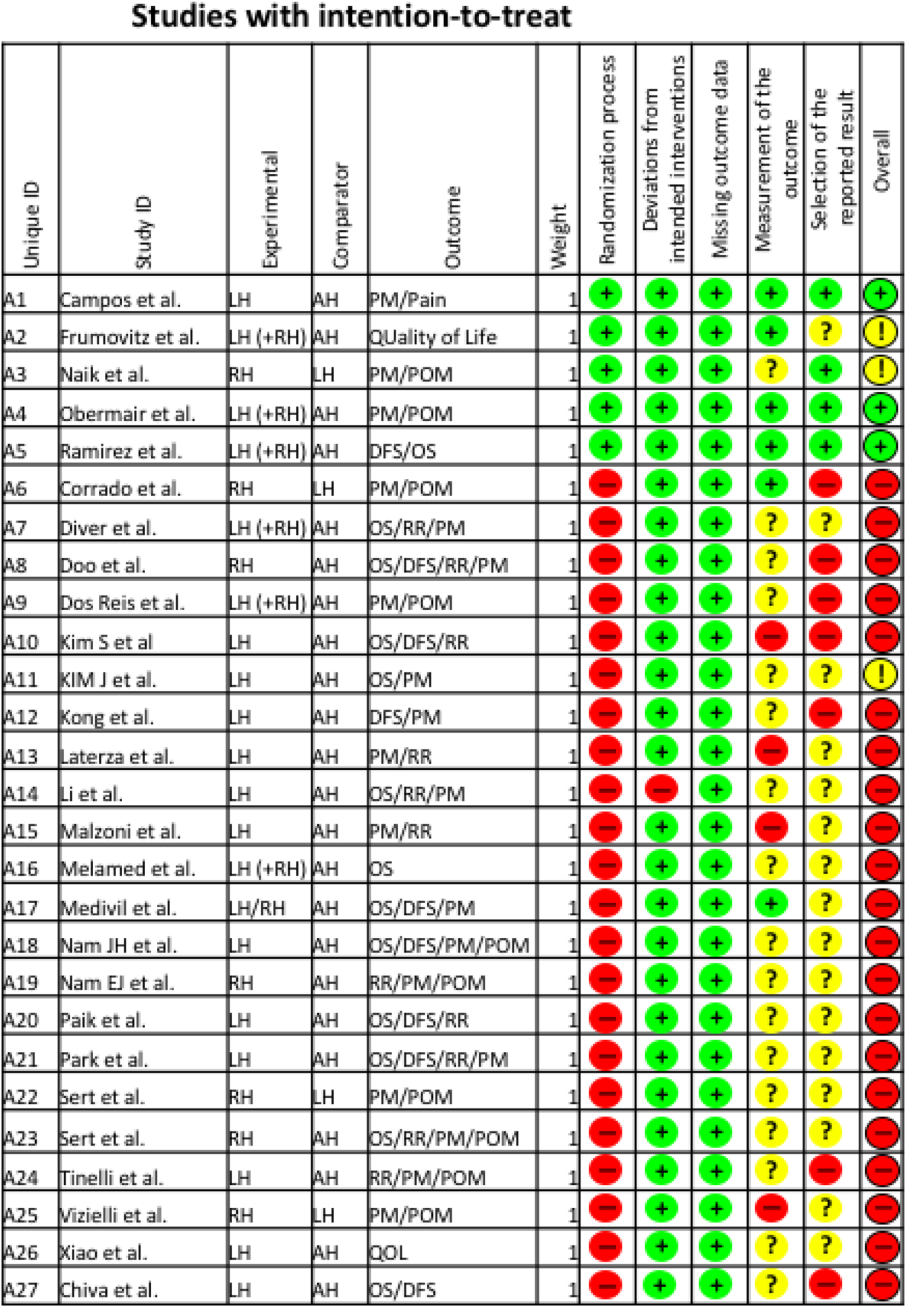
Risk of bias assessment

The updated Cochrane risk of bias tool 2 (RoB 2) was used to assess the scientific quality of the included studies [15] (Fig. 2). The quality assessments were performed by two independent researchers (JK and EG). Disagreements were resolved by consensus of all authors.

Two reviewers independently extracted the safety and effectiveness indexes into a pre-specified data extraction form and double-checked them.

### Statistical analysis

Inter-study heterogeneity was assessed using the maximum likelihood estimator with calculation of *τ*^2^ and its corresponding *p* value [16]. This *p* value indicates the probability that deviation from inter-study homogeneity can be explained by chance with a lower *p* value implying significant heterogeneity. The 95% confidence intervals (CI) were used as the summary variables for continuous outcomes and the risk rate (RR) and 95% CI for dichotomous variables.

Statistical analysis was conducted by fixed-effect models in the absence of significant heterogeneity and random-effect models in the presence of significant heterogeneity (Chi-squared test for homogeneity, *τ*^2^). Analysis was by intention to treat. Subgroup analyses were pre-specified according to the estimated risk of intervention. Using a two-sample *t* test, two-sided and right- as well as left-tailed, means were compared to receive *p* values for comparison of the subgroups.

All included studies were assessed regarding potential conflicts of interest. In all studies, the ICMJE uniform disclosure form was completed.

## Results

### Study characteristics

In total, 685 studies met the inclusion criteria and were assessed for eligibility. After removing records with no full text, duplicates and wrong study designs (e.g. reviews), 27 suitable comparative studies were included into final analysis (Fig. 1). The inclusion criteria were set analogue to the LACC trial [11]. Table 1 shows the characteristics of the 27 two-armed studies.

**Table 1:** Study characteristics

Author	Region	Publication year	Study year	FIGO Stage	Study design	Cohort	LH	RH	AH	Median follow-up (months) LH/AH
Campos et al. [24]	Brazil	2013	1999–2004	IA2–IB	RCT, single center	LH:AH	16	0	14	nn
Bogani et al. [18]	Worldwide	2020	2013–2014	IB1	Retrospecitve, multicenter	LH(+ RH):AH	291	nn	402	59/56
Corrado et al. [25]	Italy	2014	2010–2012	IA1–IIA1	Retrospective, multicenter	LH:RH	30	30	0	25
Diver et al. [26]	USA	2016	2000–2013	IA1–IIB	Retrospective, multicenter	LH(+ RH):AH	101 (71)	nn	282	61.2
Doo et al. [27]	USA	2019	2010–2016	IB1	Retrospective, multicenter	RH:AH	0	49	56	nn
Dos Reis et al. [28]	USA	2018	1990–2013	IA1, IA2, IB1, IIA1	Retrospective, singlecenter	LH(+ RH):AH	121 (50)	nn	427	10.4
Frumovitz et al. [29]	Worldwide	2020	2008–2017	IA1, IA2, IB1	RCT, multicenter	LH(+ RH):AH	319 (45)	nn	312	36
Kim et al. [30]	Korea	2019	2000–2018	IB1–IB2	Retrospective, multicenter	LH:AH	343	0	222	59.1
Kim et al. [31]	Korea	2018	2011–2014	Unknown	Retrospective, multicenter	LH:AH	3100	0	3235	nn
Kong et al. [32]	Korea	2014	2006–2013	IB–IIA	Retrospective, singlecenter	LH:AH	40	0	48	28/58
Laterza et al. [33]	Italy	2016	1997–2014	IA1, IA2, IB1, IIA1	Retrospective, singlecenter	LH:AH	82	0	68	121.2/43.5
Li et al. [34]	China	2007	1998–2005	IB–IIA	Retrospective, singlecenter	LH:AH	90	0	35	26
Malzoni et al. [4]	Italy	2009	1995–2007	IA1, IA2, IB1	Retrospective, singlecenter	LH:AH	65	0	62	52.5/71.5
Melamed et al. [17]	USA	2018	2000–2013	IA2, IB1	Retrospective, singlecenter	LH(+ RH):AH	1225 (978)	nn	1236	45
Mendivil et al. [35]	USA	2015	2009–2013	IA2–IIB	Retrospective, singecenter	LH:RH:AH	49	58	39	39
Naik et al. [36]	UK	2010	2005–2007	IB1	RCT, single center	LH:AH	8	0	7	nn
Nam et al. [3]	Korea	2011	1997–2008	IA2–IIA	Retrospective	LH:AH	263	0	263	92
Nam et al. [37]	Korea	2010	2006–2009	IA2–IIB	Retrospective, singlecenter	RH:AH	0	32	32	15.3
Obermair et al. [38]	Worldwide	2020	2008–2017	IA1, IA2, IB1	RCT, multicenter	LH(+ RH):AH	279 (41)	nn	257	6
Paik et al. [39]	Korea	2019	2000–2008	IB–IIA	Retrospective, multicenter	LH:AH	119	0	357	63.6
Park et al. [40]	Korea	2016	1997–2013	IA2–IIA	Retrospective, singlecenter	LH:AH	186	0	107	58.8
Ramirez et al. [11]	Worldwide	2018	2008–2017	IA1, IA2, IB1	RCT, multicenter	LH(+ RH):AH	319 (45)	nn	312	30
Sert et al. [41]	Norway	2007	2004–2005	IA1–IB1	Retrospective, singlecenter	LH:RH	7	7	0	14/25
Sert et al. [42]	Norway	2016	2005–2011	IA1–IB2	Retrospective, multicenter	RH:AH	0	259	232	34.6/35.2
Tinelli et al. [43]	Italy	2011	2003–2010	IA1–IIA	Retrospective, multicenter	LH:RH	76	23	0	46.5/24.5
Vizzielli et al. [44]	Italy	2016	2013–2015	IA2–IIB	Retrospective, multicenter	LH:RH	42	21	0	nn
Xiao et al. [45]	China	2016	2001–2014	IA–IIA	Retrospective, singlecenter	LH:AH	42	0	16	46.1/51.2

*AH* abdominal hysterectomy, *LH* laparoscopic hysterectomy, *nn* not named, *RCT* randomized controlled study, *RH* robotic hysterectomy

The countries the studies were conducted are the USA, Korea, China, Italy, Norway and Brazil. The publication years ranged from 2007 to 2020. In total, 16.292 patients with operative treatment of early cervical carcinoma were included. Five prospective, randomized controlled trials were included. 22 studies had a retrospective design. Use of robotic surgery among the included studies varied substantially.

In addition, all non-comparative, single-armed studies evaluating only one surgical approach were identified. Three retrospective single-center studies using total laparoscopic hysterectomy (TLH) or laparoscopically assisted vaginal hysterectomy (LAVH) were present (Table 2).

**Table 2:** Single-arm study characteristics

Author	Region	Publication year	Study year	FIGO Stage	Study design	Cohort	Number of participants	Median follow-up (months)
Kanno et al. [46]	Japan	2019	2000–2019	IA1–IB1	Retrospective, single center	LH	109	73
Köhler et al. [21]	Germany	2019	1994–2018	IA1–IIA1	Retrospective, single center	LAVH	389	99
Odetto et al. [47]	Argentina	2019	2010–2015	IA1–IB1	Retrospective, single center	LH	108	39

*LAVH* laparoscopic assisted vaginal hysterectomy, *LH* laparoscopic hysterectomy

The risk of bias assessment revealed a large overall risk of bias since most of the included studies were either not randomized or studies were not prospectively planned and the sample size had a wide range.

In cases of considerable heterogeneity between the included studies assumed (Chi-squared test for homogeneity, *τ*^2^), the results from the random effects analyses were used for meta-analysis. In cases of low heterogeneity, fixed-effects model was applied.

All included studies were assessed regarding potential conflicts of interest. In all studies, the ICMJE uniform disclosure form was completed. The detailed funding information can be found in the supplemental data.

**Fig. 3:**
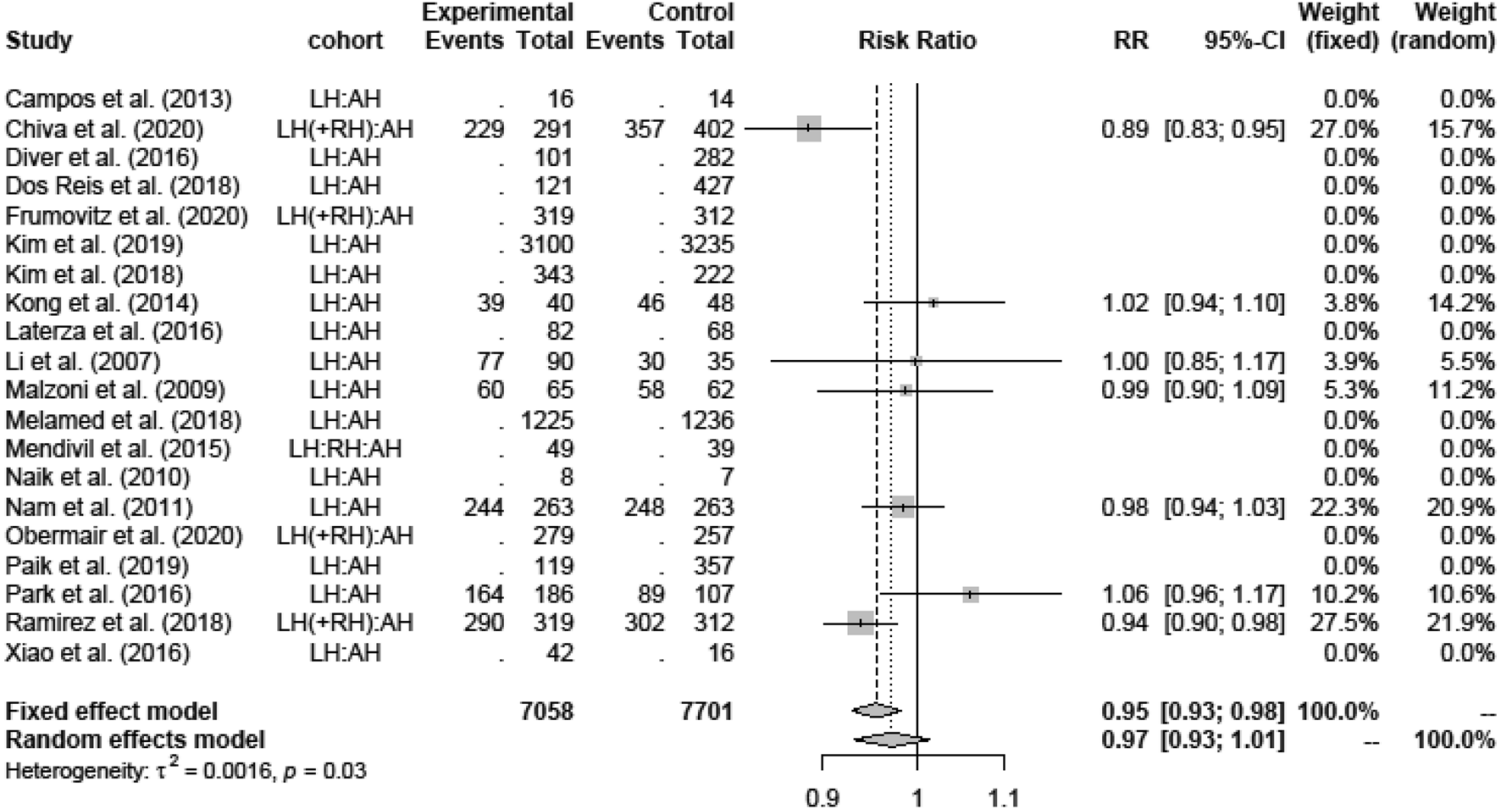
Disease-free survival LH versus AH

In the first meta-analysis, all studies comparing LH in general and AH were analyzed regarding survival rates OS and DFS. Although the RCT of Ramirez et al. [11] showed a lower DFS for LH (RR 0.97; 95%-CI [0.95; 1.00]), there was neither a difference for DFS between LH and AH in the retrospective trials, nor averaged over all studies (0.97; [0.93; 1.01]) (Fig. 3). Due to high heterogeneity, random effects model was applied.

**Fig. 4:**
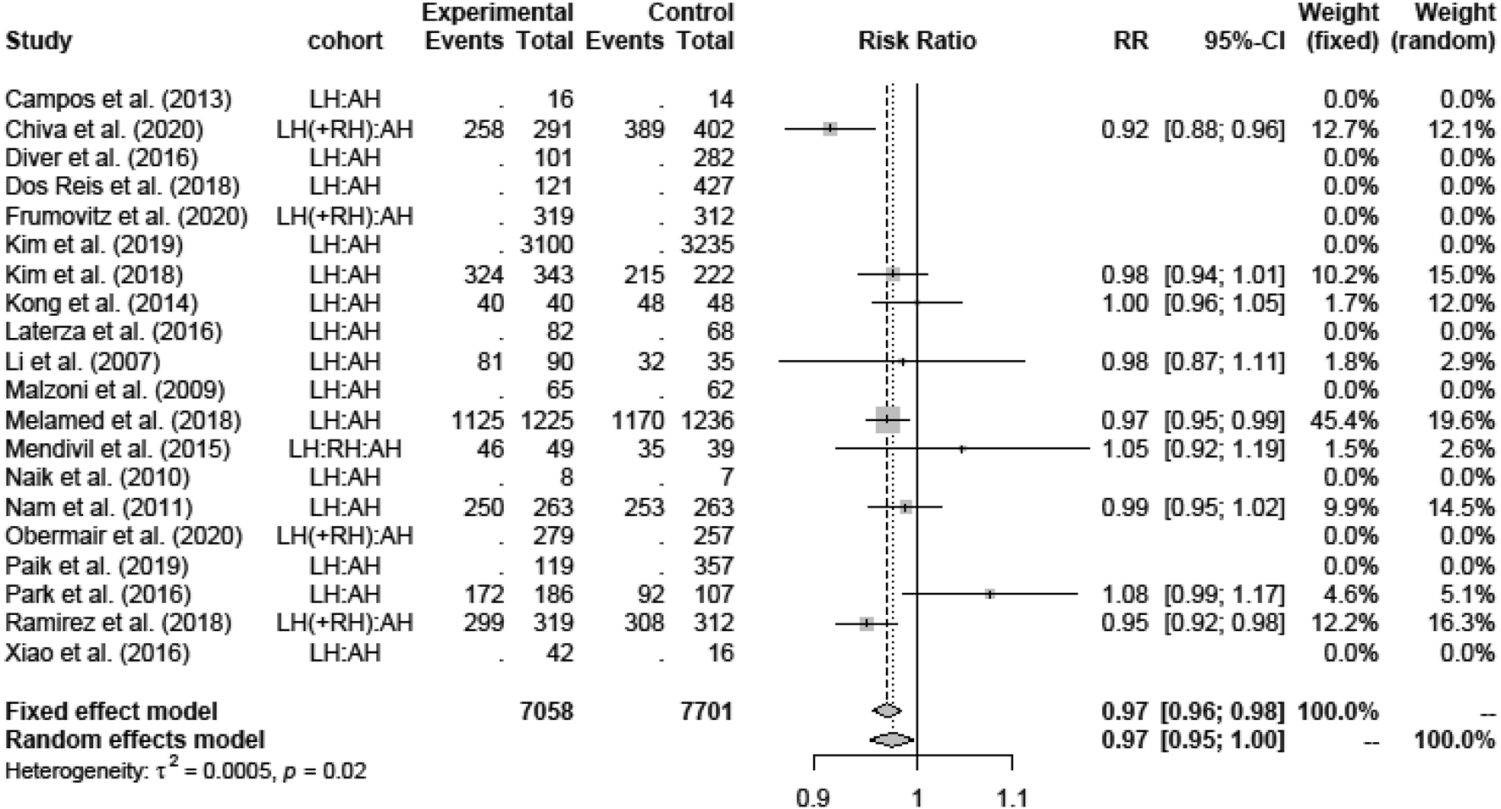
Overall-survival LH versus AH

LH showed lower OS than AH (Fig. 4) (0.97; [0.96; 0.98]). The only RCT in this group, Ramirez et al., came to the similar results as the retrospective studies of Melamed et al. [17] and Bogani et al. [18].

Four subgroups concerning the operating technique (AH, LH, uterine manipulator, colpotomy) were established and analyzed regarding DFS and OS [19] in (1) open surgery group (AH), (2) high-risk group: LH with uterine manipulator (LH + M), (3) intermediate-risk group: LH without uterine manipulator (LH − M) and (4) low-risk group: LH without uterine manipulator and with prophylactic vaginal closure (LH − M + V).

**Fig. 5:**
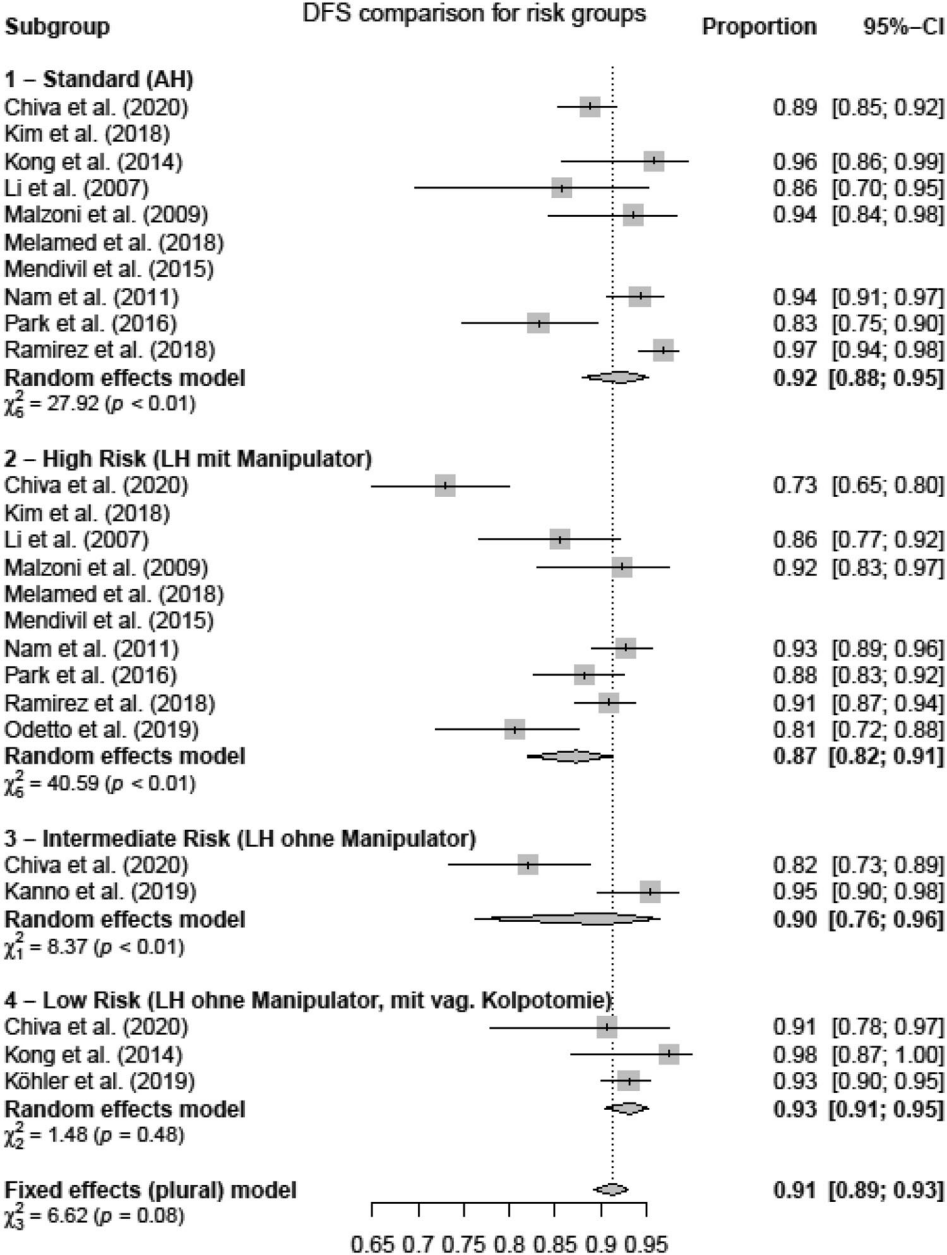
Disease-free survival risk groups

The open surgery group (subgroup 1, AH) showed a DFS of 0.92 [95%-CI 0.88; 0.95]. The high-risk group (subgroup 2) had the lowest DFS with 0.87 [0.82; 0.91], whereas in the intermediate-risk group (subgroup 3), we calculated a DFS of 0.90 [0.76; 0.96]. The highest DFS of LH subgroups was found in the low-risk group (subgroup 4) (0.91 [0.91; 0.95]) (Fig. 5).

**Fig. 6:**
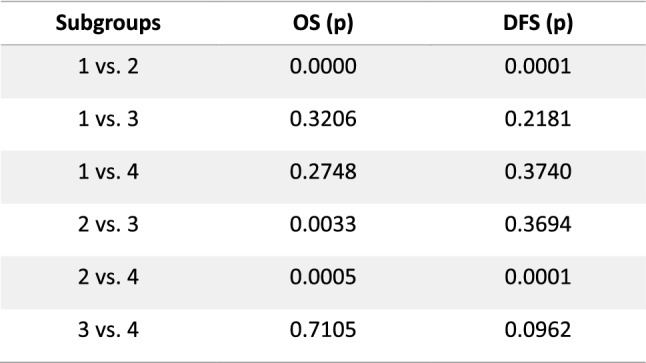
Two-sided *t* test

Figure 6 shows the *p* values for DFS of the comparison of the respective subgroups tested by two-sided *t* test. The results showed a significant superiority of subgroups 1 (AH) over 2 (LH + M) *p* = 0.0001, 4 (LH − M + V) over 2 (LH + M) *p* = 0.0001, and not significantly 4 (LH − M + V) over 3 (LH − M), *p* = 0.0962. These results show that DFS of AH is higher than LH in general, whereas omitting the risk factor uterine manipulator especially in combination with a prophylactic vaginal closure increases the rate of DFS in laparoscopy. DFS of subgroups 1 (AH) and 4 (LH − M + V) was statistically not different (*p* = 0.374).

**Fig. 7:**
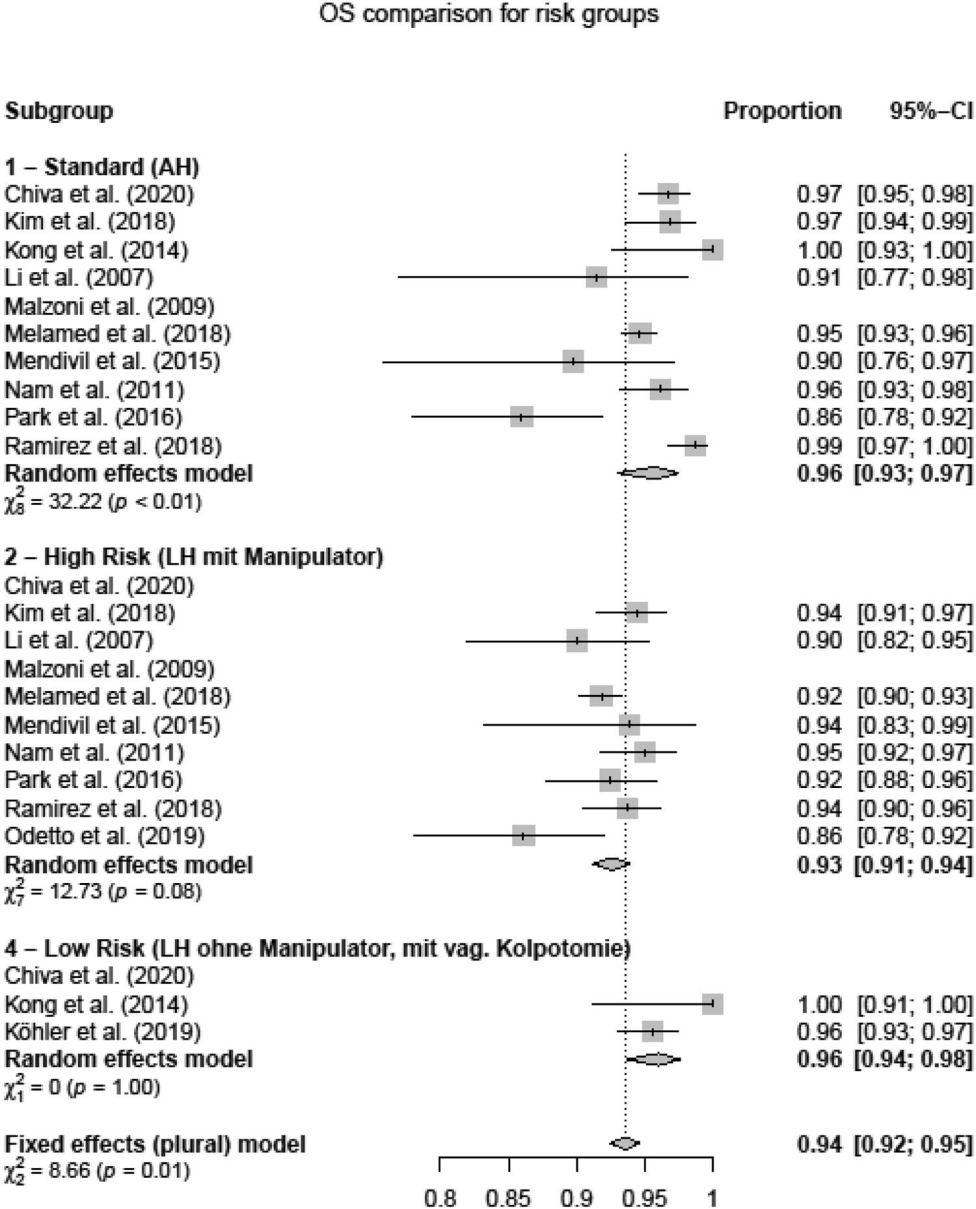
Overall-survival risk groups

The analysis of OS for the respective risk groups showed an OS of 0.96 [0.93; 0.97] in subgroup 1 (AH), 0.93 [0.91; 0.94] in high-risk group (LH + M) and 0.96 [0.94; 0.98] in low-risk group (LH − M + V) (Fig. 7). A meta-analysis of intermediate-risk group was not possible due to a lack of studies. *p* values revealed a significantly higher OS in subgroup 1 (AH) over 2 (LH + M), *p* < 0.0001. Low-risk group (subgroup 4, LH − M + V) revealed significantly higher OS than high-risk group (subgroup 2, LH + M), *p* = 0.001. Intermediate-risk group tested by two-sided *t* test (subgroup 3, LH − M) showed significantly higher OS than high-risk group (subgroup 2, LH + M), *p* = 0.0067. These results show a superiority of OS of AH and LH without manipulator/with prophylactic vaginal closure over LH with uterine manipulator. OS rates were equivalent in AH and Low-risk group (LH − M + V), *p* = 0.5496.

## Discussion

In this systematic review and meta-analysis, we not only compared the survival rates of open hysterectomy and laparoscopic hysterectomy, but also the results of risk groups including uterine manipulators and prophylactic vaginal suture. To our knowledge, this is the first meta-analysis stratifying risk groups subject to operation techniques (AH, LH, uterine manipulator and prophylactic vaginal suture).

Our analysis showed a significantly higher DFS and OS in the open surgery group of 92 and 96%, respectively, compared to the minimally invasive group. These results are in line with the outcomes of the LACC trial [11] as well as a recently published meta-analysis by Nitecki et al. [12]. This meta-analysis of 15 observational studies revealed a 71% higher hazard of recurrence and death in the minimally invasive radical hysterectomy group compared to the open surgery cohort. As a strength of their meta-analysis, the authors described their various methods used to minimize confounding, e.g. by demographic factors, tumor stage or size. Unfortunately, the authors did not evaluate the association of different surgical techniques with survival after laparoscopic radical hysterectomy. However, the missing standardization of laparoscopic surgical techniques is one of the main critics to all recently published studies. The greatest concern regards the risk of intraoperative tumor dissemination during surgery which appears to be promoted by the use of a uterine manipulator and intracorporal colpotomy.

Most surgical groups use uterine manipulators which may ease the handling of the uterus and improve the visualization but disrupt tumor integrity. Moreover, the vaginal cuff is opened laparoscopically above the manipulator rim after parametrial resection. This maneuver (intracorporal colpotomy), however, may spread tumor cells within the peritoneal cavity exposed to circulating carbon dioxide. The principle of tumor cell dissemination during intracorporal colpotomy was visualized by Klapdor et al. [20].

Concerning the possible tumor cell dissemination during intracorporal colpotomy, Köhler et al. [21] pointed out the importance of avoiding tumor cell dissemination not only by omitting the uterine manipulator but by creating a tumor-covering vaginal cuff transvaginally. In their single-arm study, they reached high 10-year OS and DFS rates (93.1 and 95.8%, respectively). These results are supported by the publication of Kong et al., who described a HR of 3.059 (95% CI 1.176–7.958; *p* = 0.022) [22] for risk of recurrence when intracorporal instead of vaginal colpotomy was performed.

Similarly, the use of a uterus manipulator might spill tumor cells into the abdominal cavity or blood and lymph vessels. Bogani et al. [18] found a hazard ratio of 2.76 for relapse for the use of uterine manipulators in minimally invasive surgery. Moreover, the DFS of patients not treated with manipulators showed comparable DFS as the open surgery group. In addition, patients that underwent minimally invasive surgery with additional protective vaginal closure had similar, but not significant, relapse rates to those in the open surgery group (HR 0.63, p < 0.52). Interestingly, in a recent study by Nica et al. use of an intra-uterine manipulator was not an independent factor associated with recurrence [23]. However, this study cannot be taken into account since tumors > 40 mm were included and residual tumor was present in a very high percentage of 68%.

To further investigate this hypothesis, we created and compared the four subgroups as described before. In the low-risk group (subgroup 4, no manipulator, prophylactic vaginal closure), survival rates comparable to the AH group (DFS 91 vs. 92%, *p* = 0.37 and OS 94 vs 96%, *p* = 0.55) were found. Furthermore, as shown in the intermediate-risk group, the use of a uterine manipulator and intracorporal colpotomy was associated with decreased DFS compared to vaginal colpotomy group (*p* = 0.0962).

In contrast to the meta-analysis by Nitecki et al., we did not stratify the groups according to confounding factors to have enough patients for the subgroup analysis since these factors were not assessable in most studies. Our review also has limitations, which mainly involve the heterogenous and mostly retrospective study designs. There are no prospective studies systematically evaluating the effects of these surgical techniques on the outcome. Second, the sometimes very small patient samples could lead to a bias dependent on the surgeon’s abilities in the field of especially newer techniques, such as the robotic hysterectomy. A strict separation between robotic and classic laparoscopic techniques was not feasible.

Keeping the limitations of our analysis in mind, the results of our meta-analysis do support the hypothesis that laparoscopic radical hysterectomy when executed with safety measures against intraoperative tumor spillage, such as the avoidance of uterine manipulators and conduction of vaginal colpotomy, appears to be associated with comparable disease-free and overall survival rates as abdominal surgery. These results have to be considered when designing additional prospective studies on this topic.

## Conclusion

Further prospective studies with standardized surgical techniques are pending to investigate and improve survival rates of minimally invasive approaches.

## Supplementary Information

Below is the link to the electronic supplementary material.

Supplementary file1 (DOCX 153 kb)

## Data Availability

Not applicable.
