# Supplementary figures and images for: Protective operative techniques in radical hysterectomy in early cervical carcinoma and their influence on disease-free and overall survival: a systematic review and meta-analysis of risk groups
Source: Arch Gynecol Obstet. 2021 May 22;304(3):577–87. doi: 10.1007/s00404-021-06082-y (PMC8325671; doi:10.1007/s00404-021-06082-y)

Supplemental: Medline (Ovid) Session results


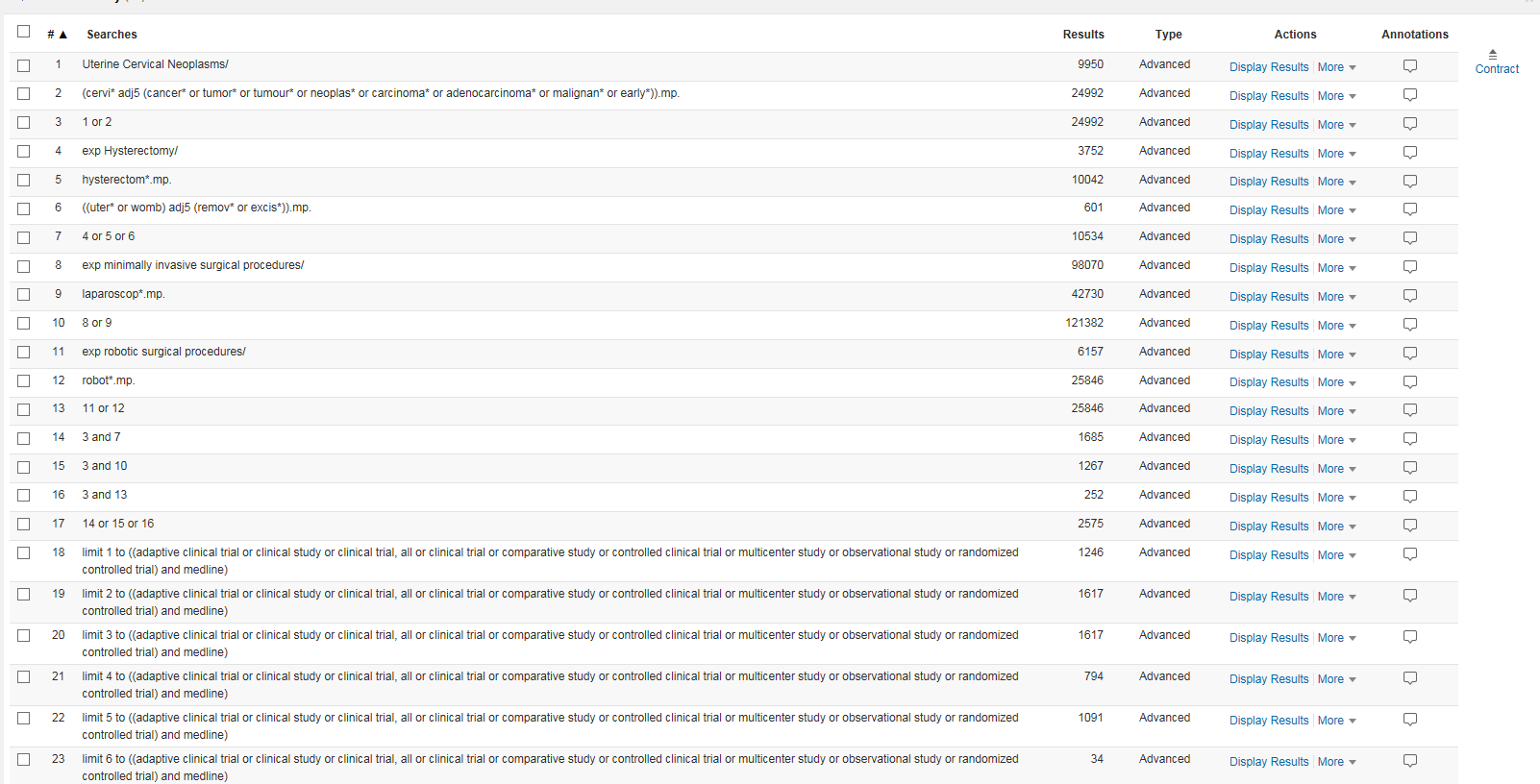


Supplemental: Embase Session results


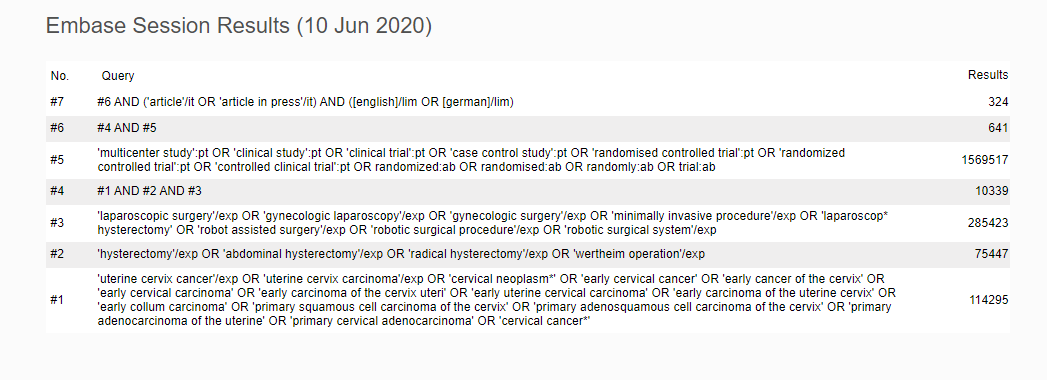

Supplement: Supplementary file 1 — Supplementary file1 (DOCX 153 kb) [file 404_2021_6082_MOESM1_ESM.docx]
